# Neuropsychological Functioning in Transgender Adults: Evidence from a University-Based Gender Identity Clinic in Türkiye

**DOI:** 10.3390/brainsci16090918

**Published:** 2026-08-28

**Authors:** Hanife Yılmaz Abaylı, Ezgi Şişman, Fatma Seher Kocaayan, Aslıhan Polat

**Affiliations:** 1 Department of Psychiatry, Eyüpsultan State Hospital, 34050 İstanbul, Türkiye; 2 Department of Psychiatry, Kocaeli City Hospital, 41060 Kocaeli, Türkiye; drezgisisman@gmail.com; 3 Department of Psychiatry, Sakarya Training and Research Hospital, 54100 Sakarya, Türkiye; seherkocaayan@gmail.com; 4 Department of Psychiatry, Faculty of Medicine, Kocaeli University, 41001 Kocaeli, Türkiye; aslihan.polat@kocaeli.edu.tr

**Keywords:** gender incongruence, gender identity, neuropsychological assessment, cognitive functioning, transgender health, minority stress

## Abstract

**Highlights:**

**What are the main findings?**

- Among 81 adults at a gender identity clinic in Türkiye, group differences emerged primarily in visuospatial and executive-control measures, while processing speed, working memory, and verbal fluency showed substantial overlap.
- Both transgender groups performed lower than cisgender groups on visuospatial measures, whereas executive-control patterns followed a different ordering; the distributions overlapped substantially and should not be read as indicating correspondence between cognitive performance and either gender identity or sex assigned at birth; no clear association with hormone therapy status was detected in this small sample.

**What are the implications of the main findings?**

- Cognitive test performance is not a marker of gender identity. Most domains did not distinguish the groups, and scores overlapped substantially even where group-level differences were observed.
- Findings from this non-Western clinical sample support context-sensitive interpretation rather than fixed-trait or deficit models and highlight the need for more inclusive neuropsychological reference data for transgender populations.

**Abstract:**

Background: Neurocognitive functioning in transgender adults remains understudied and is predominantly derived from Western samples, often treated as universally applicable. Evidence from non-Western contexts is limited, despite the potential influence of structural stigma and minority stress on cognition and research participation. Türkiye represents a sociopolitical setting where transgender individuals face significant barriers to healthcare and research. To date, no study has systematically examined the neuropsychological profile of transgender adults in Türkiye using a four-group design. Methods: This retrospective, cross-sectional study was conducted at a multidisciplinary university-based gender identity clinic. Eighty-one adults were included: transgender women (*n* = 14), transgender men (*n* = 39), cisgender women (*n* = 13), and cisgender men (*n* = 15), with cisgender groups matched for age and education. Participants completed a standardized neuropsychological battery assessing visuospatial abilities, executive functions/processing speed, working memory, and verbal fluency. Group differences were analyzed using nonparametric methods, with additional aggregated transgender–cisgender comparisons. Results: Group differences were observed in visuospatial functioning (Judgment of Line Orientation, Rey–Osterrieth Complex Figure; all *p* ≤ 0.05) and executive control (Trail Making Test Part B, *p* = 0.048; B–A difference, *p* = 0.014). Group-level differences were domain-specific: both transgender groups scored below both cisgender groups on the visuospatial measures, whereas the ordering on executive-control measures differed, and distributions overlapped substantially throughout. Processing speed, working memory, and verbal fluency did not differ significantly (*p* > 0.05). No clear association between hormone therapy status and performance was detected in this small sample. Conclusions: The observed distributions were domain-specific and substantially overlapping, and should not be interpreted as indicating correspondence between cognitive performance and either gender identity or sex assigned at birth. Differences were domain-specific, with substantial overlap across groups. Findings support a context-sensitive, non-pathologizing interpretation emphasizing sociocultural factors rather than inherent deficits. Longitudinal and cross-cultural studies incorporating psychosocial variables are needed.

## 1. Introduction

Transgender and gender diverse (TGD) individuals are those whose gender identity and/or gender expression differs from social expectations associated with sex assigned at birth (SAAB). In contemporary diagnostic frameworks, this incongruence is conceptualized in a non-pathologizing manner: while the DSM-5-TR uses the term gender dysphoria to denote clinically significant distress that may accompany this incongruence, the ICD-11 has relocated gender incongruence to conditions related to sexual health. This shift reflects an international effort to reduce stigma while maintaining access to gender-affirming care and essential health services [1,2,3]. Despite increasing clinical and scientific attention, etiological pathways and neurobiological correlates associated with gender incongruence remain incompletely understood and are best conceptualized as multifactorial, encompassing neurodevelopmental, endocrine, and psychosocial processes [4,5,6]. Neuroimaging studies have reported group-level differences in brain structure and function among transgender individuals, including variations in cortical thickness, gray and white matter volumes, and connectivity patterns [7]. Importantly, these findings are heterogeneous, characterized by substantial within-group variability, and do not constitute diagnostic or deterministic markers [5,6,8,9].

In contrast to the expanding neuroimaging literature, neurocognitive research in transgender adults remains limited and largely Western-centric [10,11]. Most existing studies have been conducted in Western Europe and North America, often implicitly treating cognitive profiles derived from these contexts as universal reference points [10,11]. This tendency risks overlooking how sociocultural, linguistic, and political environments shape neuropsychological performance, particularly in non-Western and more conservative societies [12,13,14]. Neurocognitive functioning in transgender individuals must therefore be interpreted within broader social and structural contexts. Minority Stress Theory posits that chronic exposure to stigma, discrimination, and social exclusion generates sustained psychophysiological stress, which can deplete cognitive resources over time and disproportionately affect domains such as executive control, attentional flexibility, and complex visuospatial processing [15,16,17]. Under such conditions, neuropsychological test performance may reflect stress-related cognitive load rather than necessarily indicating stable or intrinsic cognitive characteristics. However, as minority stress was not directly measured in this study, these interpretations should be considered as theoretically informed hypotheses rather than empirically tested mechanisms.

Türkiye represents a sociopolitical context in which these dynamics are particularly salient. Empirical studies document high levels of perceived discrimination against transgender individuals in education, employment, and healthcare, alongside significant barriers to accessing gender-affirming services [18,19,20]. Beyond interpersonal stigma, recent reports by international human-rights organizations describe a restrictive political climate characterized by hostile official rhetoric toward LGBTI+ communities, limitations on civil society activity, and growing pressures on academic freedom [21,22]. In such an environment, transgender individuals are often required not only to navigate daily discrimination but also to actively defend their identities within a broader atmosphere of political scrutiny. These conditions may influence both participation in research and performance during evaluative settings, including neuropsychological assessment.

To our knowledge, no prior study has systematically examined the neuropsychological profile of transgender adults in Türkiye. The absence of such data constitutes a critical gap in the international literature, particularly given the influence of language, education, and sociopolitical context on neuropsychological assessment outcomes. Generating context-specific evidence is therefore essential not only for scientific completeness but also as a form of documentation of health equity in settings where transgender health research itself may face structural barriers.

The present study was conducted in a multidisciplinary, university-based gender-affirming care clinic in Türkiye, integrating psychiatric, psychological, endocrinological, and surgical services in line with international standards of care [3,23]. Using a standardized neuropsychological battery and a four-group analytic framework (transgender women, transgender men, cisgender women, cisgender men), we aimed to characterize cognitive functioning across visuospatial abilities, executive functions, working memory, and verbal fluency.

Cisgender participants were included as a contextual reference group rather than as a normative benchmark.

Rather than testing whether cognitive performance is better explained by gender identity or by sex assigned at birth—a framing that risks treating cognitive profiles as markers of identity—we approached the four-group comparison descriptively. We therefore expected that any group-level differences would be domain-specific and accompanied by substantial overlap between groups, and we treated sociocultural context as integral to their interpretation. By providing the first comprehensive neuropsychological data from an adult transgender sample in Türkiye, this study aims to contribute to a culturally informed and non-pathologizing understanding of cognitive functioning in transgender populations.

## 2. Materials and Methods

### 2.1. Study Design and Setting

This study was designed as a retrospective, cross-sectional analysis of neuropsychological assessment data obtained during routine clinical care. Data were collected at the Gender Identity Clinic of Kocaeli University Faculty of Medicine, a tertiary university hospital providing multidisciplinary gender-affirming care in line with international standards.

The clinic operates within an integrated care model that includes psychiatry, psychology/neuropsychology, endocrinology, and surgical specialties. Clinical assessment and follow-up are conducted in accordance with the World Professional Association for Transgender Health (WPATH) Standards of Care, Version 8, and the European Network for the Investigation of Gender Incongruence (ENIGI) framework, ensuring standardized, ethically grounded, and affirming care pathways.

As part of routine clinical practice, individuals applying to the clinic are informed from the outset that their anonymized clinical data may be used for scientific purposes aimed at improving knowledge and services in the field of gender incongruence. Written informed consent is obtained in accordance with institutional and ethical regulations, and confidentiality is strictly maintained [23].

### 2.2. Participants

#### 2.2.1. Transgender Group

The transgender sample consisted of adults aged 18 years and older who applied to the clinic between 2020 and 2024 for gender-affirming evaluation and were diagnosed with gender dysphoria according to DSM-5-TR criteria by two experienced psychiatrists. All transgender participants identified with binary gender identities (transgender women or transgender men) and reported heterosexual orientation in routine clinical records. For the purposes of this study, we focused on this relatively homogeneous clinical subgroup to reduce heterogeneity related to sexual orientation and variations in clinical presentation that could confound neurocognitive comparisons. This profile also reflects the group that most commonly seeks gender reassignment–related clinical services within the current legal and medical framework in Türkiye. Clinical records further indicated an early-onset developmental history of gender dysphoria in this sample, which additionally reduced clinical heterogeneity for the present analyses.

Neuropsychological assessments are routinely conducted in this clinic as part of comprehensive psychiatric evaluation and longitudinal follow-up. Testing appointments were scheduled when participants attended the clinic in relation to the ongoing group psychotherapy program for transgender individuals, which constitutes a standard component of psychosocial care in this setting.

#### 2.2.2. Cisgender Control Group

The cisgender control group was recruited using a convenience sampling method from relatives of patients attending the internal medicine outpatient clinics of the same university hospital. Controls self-identified as cisgender women or cisgender men, reported no history of gender incongruence, and agreed to participate voluntarily.

This recruitment strategy was chosen to maximize sociocultural and socioeconomic comparability between transgender and cisgender participants by sampling from the same hospital environment and geographic catchment area. The control group was frequency-matched to the transgender group by age and total years of education, thereby minimizing potential confounding effects of these variables on neuropsychological performance.

### 2.3. Inclusion and Exclusion Criteria

Inclusion criteria for all participants were:

- Age ≥ 18 years
- Sufficient literacy to complete neuropsychological testing

Exclusion criteria were stringently applied to reduce the influence of conditions known to affect cognitive functioning and included:

- Psychotic disorders
- Current Major Depressive Disorder meeting DSM-5-TR criteria
- Neurodegenerative disorders or other neurological conditions with potential cognitive impact
- Intellectual disability
- Known intracranial pathology
- Pregnancy or breastfeeding
- Illiteracy

By explicitly excluding individuals with psychosis, neurodegenerative disorders, and current major depressive disorder, the study aimed to ensure that observed neuropsychological findings were independent of severe psychiatric or neurological conditions known to compromise cognitive performance. Systemic conditions with potential cognitive relevance—including hypertension, diabetes mellitus, cerebrovascular disease, human immunodeficiency virus infection, and diagnosed sleep disorders—were screened for during the clinical assessment, and none were present in any participant in either the transgender or the cisgender groups; the groups were therefore comparable with respect to these conditions.

Following application of inclusion and exclusion criteria, data from a total of 81 participants were included in the analysis: 14 transgender women, 39 transgender men, 13 cisgender women, and 15 cisgender men. Transgender and cisgender groups did not differ significantly in age or total years of education.

### 2.4. Neuropsychological Assessment Procedure

All neuropsychological assessments were administered by a single experienced clinical psychologist trained in neuropsychological testing. The psychologist was independent of the gender clinic team and did not participate in clinical decision-making regarding gender-affirming treatment, thereby minimizing procedural and expectancy bias.

Neuropsychological testing was conducted as part of the clinic’s routine assessment pathway, rather than as a stand-alone research procedure. Testing sessions were typically scheduled when transgender participants attended the clinic in relation to the ongoing group psychotherapy program, enhancing the ecological validity of the findings.

Testing sessions were conducted in a standardized, quiet clinical environment and lasted approximately 60–90 min per participant. The same fixed order of tests was used for all participants to ensure procedural consistency. To enhance data integrity and reduce potential bias, an independent clinical psychologist verified test scoring and electronic data entry against the original records while blinded to participants’ transgender versus cisgender status.

Order of test administration:

(1) Trail Making Test (Parts A and B)
(2) Rey–Osterrieth Complex Figure Test—copy condition
(3) Verbal Fluency Tests (semantic fluency: animal naming; phonemic fluency: K, A, and S letters)
(4) Rey–Osterrieth Complex Figure Test—immediate recall
(5) Rey–Osterrieth Complex Figure Test—delayed recall
(6) WAIS-III Digit Span Test (forward and backward)
(7) Benton Judgment of Line Orientation Test

#### Neuropsychological Measures

The standardized test battery assessed multiple cognitive domains:

- Judgment of Line Orientation Test (JLO): assessment of visuospatial perception and spatial orientation without motor coordination demands.
- Rey–Osterrieth Complex Figure Test (ROCF): evaluation of visuoconstructional ability (copy), visual memory (immediate and delayed recall), and recognition.
- Trail Making Test (TMT) Parts A and B: assessment of processing speed and visual attention (Part A), and executive functioning including cognitive flexibility and set-shifting (Part B); the B–A difference was calculated.
- WAIS-III Digit Span Test: evaluation of attention (forward span) and working memory (backward span).
- Verbal Fluency Tests: semantic fluency (animal naming) and phonemic fluency (K, A, S letters), each administered for 60 s.

All tests were administered and scored according to standardized procedures.

### 2.5. Hormonal and Sociodemographic Variables

Age, total years of education, and handedness were recorded for all participants. For transgender participants, hormonal status was evaluated as part of routine multidisciplinary care. Available laboratory data on serum hormone levels (total testosterone and estradiol), gender-affirming hormone therapy (GAHT) status at the time of assessment (receiving vs. not receiving), and duration of hormone treatment (months) were extracted from medical records for descriptive and exploratory analyses. Serum total testosterone and estradiol concentrations were also obtained for cisgender participants as part of the same assessment pathway. Reproductive status was also ascertained in cisgender women: all were premenopausal, and none reported oral contraceptive use.

### 2.6. Statistical Analysis

Statistical analyses were performed using IBM SPSS Statistics for Windows, version 22.0 (IBM Corp., Armonk, NY, USA). Descriptive statistics included mean, standard deviation, median, minimum, and maximum values. Normality of distributions was assessed using the Kolmogorov–Smirnov test. As most variables did not meet normality assumptions, non-parametric statistical tests were applied.

Between-group comparisons were conducted using the Mann–Whitney U test for two-group comparisons and the Kruskal–Wallis test for multiple-group comparisons. When appropriate, Dunn’s post-hoc tests with Bonferroni correction were performed.

Categorical variables were analyzed using chi-square tests. Exploratory associations between neuropsychological test scores and continuous clinical variables were examined using Spearman rank correlation coefficients. Statistical significance was set at *p* < 0.05 (two-tailed). Given the number of statistical comparisons performed, results should be interpreted with caution due to the potential for Type I error inflation. Given the modest size of the four subgroups, all group comparisons should be regarded as descriptive and exploratory rather than confirmatory: the study was not powered to detect small-to-moderate effects, inferential tests are reported to characterise the observed distributions rather than to establish population-level differences, and no correction for multiplicity across domains was applied.

### 2.7. Ethical Considerations

The study protocol was approved by the Non-Interventional Clinical Research Ethics Committee of Kocaeli University (Approval No. GOKAEK-2024/14.01; Project No. 2024/158). All procedures complied with the Declaration of Helsinki and Good Clinical Practice guidelines. Only anonymized retrospective data were used, and participation or non-participation in the study had no influence on the clinical care provided to any individual.

## 3. Results

### 3.1. Sociodemographic and Clinical Characteristics

A total of 81 participants were included in the analysis: 14 transgender women (TW), 39 transgender men (TM), 13 cisgender women (CW), and 15 cisgender men (CM). Sociodemographic characteristics by gender group are presented in Table 1.

No significant differences were observed across the four groups in total years of education or handedness distribution (all *p* > 0.05). A significant difference in age was detected across groups (Kruskal–Wallis *p* = 0.022), driven by younger age among transgender women compared with transgender men; age distributions otherwise overlapped substantially across groups.

Serum hormone levels (total testosterone and estradiol) were available for transgender participants and are presented descriptively. Given the observed age difference across groups, findings should be interpreted cautiously, as age may have influenced neuropsychological performance despite the overall overlap in distributions.

### 3.2. Visuospatial Functions

Significant group differences were observed across all visuospatial measures (Table 2).

On the Judgment of Line Orientation Test, performance differed significantly across groups (*p* < 0.001). Cisgender men demonstrated the highest mean scores, whereas both transgender groups showed lower performance relative to cisgender participants.

For the Rey–Osterrieth Complex Figure Test, significant group effects were observed for copy, immediate recall, delayed recall, and recognition indices (all *p* ≤ 0.05).

Cisgender men consistently achieved the highest scores, followed by cisgender women. Both transgender groups demonstrated lower performance relative to cisgender participants.

### 3.3. Executive Functions and Processing Speed

Group differences were observed for executive function–dependent measures of the Trail Making Test (Table 2).

Completion time for TMT-A did not differ significantly across groups (*p* > 0.05). In contrast, significant group effects were observed for TMT-B completion time (*p* = 0.048) and, more robustly, for the B–A difference score (*p* = 0.014). The B–A difference, which more specifically reflects cognitive flexibility and executive control independent of basic processing speed, showed the largest numerical differences across groups. Cisgender women exhibited the fastest performance, followed by cisgender men, whereas both transgender groups showed longer completion times, with transgender women demonstrating the highest B–A difference values.

### 3.4. Working Memory and Attention

No statistically significant differences were observed across the four gender groups for forward digit span, backward digit span, or total digit span scores (Table 2).

Descriptively, transgender men demonstrated the highest median backward digit span scores, followed by cisgender men, cisgender women, and transgender women.

### 3.5. Verbal Fluency

No statistically significant differences were observed across groups for semantic or phonemic verbal fluency measures (Table 2).

Cisgender men demonstrated the highest semantic fluency scores, whereas transgender women and transgender men showed comparable intermediate performance.

### 3.6. Contextual Transgender–Cisgender Comparison

For contextual reference, aggregated comparisons between transgender and cisgender participants were conducted. Considered as a single group, transgender participants had lower average scores on the visuospatial measures and longer average completion times on the executive-function measures than cisgender participants, with substantial overlap between the group distributions. As with the four-group comparisons, this aggregated analysis is descriptive and exploratory and is reported for context only.

### 3.7. Exploratory Analyses: Hormone Status and Correlations

Among transgender participants, 11 of 14 transgender women and 21 of 39 transgender men were receiving GAHT at the time of assessment. GAHT duration and serum hormone concentrations (total testosterone and estradiol) are summarized in Table 3. As expected, testosterone concentrations were higher among transgender men receiving GAHT than among those not receiving GAHT, whereas testosterone concentrations were lower among transgender women receiving GAHT than among those not receiving GAHT. Estradiol concentrations showed substantial within-group variability in both transgender subgroups. In the present sample, mean GAHT duration was 17.0 ± 9.2 months in transgender women and 27.7 ± 19.4 months in transgender men. For reference, serum concentrations in the cisgender comparison groups were within expected adult ranges: total testosterone 4.35 ± 0.71 µg/L and estradiol 29.1 ± 7.0 pg/mL in cisgender men, and total testosterone 0.23 ± 0.14 µg/L and estradiol 58.3 ± 34.2 pg/mL in cisgender women.

Exploratory analyses examined whether GAHT status (receiving vs. not receiving), GAHT duration, and serum hormone concentrations were associated with neuropsychological performance within the transgender sample. Neuropsychological test scores did not differ significantly between transgender participants receiving GAHT and those not receiving GAHT across cognitive domains (all *p* > 0.05). In Spearman correlation analyses, GAHT duration and estradiol concentrations were not significantly associated with neuropsychological outcomes (all *p* > 0.05). Total testosterone showed a weak positive association with completion time for ROCF delayed recall (rs = 0.254, *p* = 0.022), indicating longer completion time at higher testosterone levels; no other hormone–cognition correlations reached statistical significance. Given the number of exploratory comparisons and the small size of some subgroups (particularly transgender women not receiving GAHT), these findings should be interpreted cautiously and do not support causal inference.

## 4. Discussion

### 4.1. Principal Findings and Contribution

This study presents the first comprehensive neuropsychological characterization of transgender adults in Türkiye using a four-group design (transgender women, transgender men, cisgender women, cisgender men).

A major strength of this study is the single-site, single-language nature of the dataset. Unlike many neurocognitive studies in transgender populations that rely on pooling modest samples across multiple countries or centers—thereby introducing heterogeneity in language, education systems, clinical pathways, and test administration—our participants were evaluated within the same clinical setting, cultural context, and linguistic environment. All assessments were administered in a standardized manner by a single trained clinical psychologist using an identical protocol. This single-site, single- examiner design substantially reduces between-center procedural variability and interrater effects, strengthening internal validity and supporting a more internally consistent characterization of neuropsychological performance in a non-Western clinical cohort.

By foregrounding four-group comparisons rather than relying solely on aggregated transgender–cisgender contrasts, the study addresses a central methodological limitation repeatedly emphasized in the literature—namely, the tendency to oversimplify transgender neurocognition through binary analytic frames derived largely from Western samples [13,24].

Across cognitive domains, the most consistent group differences emerged in visuospatial and executive function–dependent measures, whereas working memory and verbal fluency showed substantial overlap across groups. Importantly, these distributions are domain-specific and substantially overlapping, and should not be interpreted as indicating correspondence between cognitive performance and either gender identity or SAAB. Instead, the findings suggest domain-specific configurations shaped by a combination of sociocultural context, stress exposure, and assessment conditions.

### 4.2. Why Framing Performance as Aligning with Identity or Sex Assigned at Birth Is Problematic

A longstanding line of research in transgender neurocognition has sought to determine whether performance on so-called “sex-sensitive” tasks corresponds more closely to gender identity or to SAAB. We regard this framing as problematic, for the reasons set out below. In interpreting these findings, it is important to consider that the battery deliberately included domains that have been discussed as “gender-sensitive” in cisgender normative research (e.g., visuospatial perception/visuoconstruction such as JLO and ROCF), alongside domains that tend to show smaller, inconsistent, or task-dependent sex/gender differences (e.g., digit span) and domains where modest female advantages are sometimes reported (e.g., verbal fluency). This design was intended to avoid overinterpreting any single domain as a general marker of sex or gender and to support domain-specific inference. Early studies—particularly those conducted in untreated or pre-hormonal samples—often concluded that cognitive performance aligned more closely with biological sex than with experienced gender [25]. However, subsequent work has shown that such conclusions are highly contingent on task selection, analytic strategy, and sociocultural context [24,26].

Descriptively, both transgender groups scored below both cisgender groups on every visuospatial measure that differed between groups, while the ordering within the transgender and within the cisgender pairs varied from measure to measure and the distributions overlapped substantially. A broader pattern of brain-health disparities affecting sexual and gender minority groups has been reported in the wider literature: in a US population study of 393,041 adults, sexual and gender minority participants had higher odds of an adverse brain-health composite of stroke, dementia, and late-life depression than cisgender heterosexual participants [27]. This association persisted after adjustment for cardiovascular risk factors, comorbidities, and neighbourhood deprivation; the authors called for further research into the structural causes of this inequity. Our findings are compatible with, but do not establish, the view that disparities of this kind reflect the social and structural exposures associated with belonging to a minoritized community; the present study did not measure those exposures. Small average differences between women and men have been reported in cisgender populations [28,29], but the present distributions do not map onto that literature in any consistent direction, and we do not interpret them as evidence about which category cognitive performance resembles. The ordering within the transgender pair and within the cisgender pair likewise varied from measure to measure, and no single group-level ordering held across all measures.

Taken together, these findings reinforce contemporary perspectives emphasizing that sex/gender-related differences in cognition are typically small, overlapping, and highly context-dependent rather than categorical or deterministic [26,29]. Our results therefore caution against interpretive frameworks that privilege either gender identity or SAAB as a singular explanatory axis. It is also important to consider the expected domain characteristics of the neuropsychological measures used. In cisgender populations, visuospatial tasks such as the Judgment of Line Orientation Test and the Rey–Osterrieth Complex Figure Test have historically shown modest average advantages in men, whereas verbal fluency tasks sometimes demonstrate modest advantages in women. In contrast, digit span measures are generally considered relatively gender-neutral in meta-analytic studies. The present pattern—clearer differences in visuospatial and executive domains but substantial overlap in digit span and verbal fluency—therefore aligns with the domain-specific nature of cognitive sex differences rather than suggesting a uniform gender-typical cognitive profile.

Accordingly, we do not regard this framing as an appropriate basis for interpreting cognitive performance in real-world clinical cohorts. Neurocognitive performance in transgender adults is more plausibly shaped by the interaction of domain demands, developmental and hormonal factors, and sociocultural stress exposure, rather than mapping neatly onto a single categorical axis. We would go further: posing the question in this form is itself problematic. Asking which category a cognitive profile best matches implies that neurocognitive measures could serve to characterise or adjudicate identity, a use for which they have neither validity nor legitimacy. Sex-associated differences in cognition are increasingly understood as the product of interactions between hormonal state and social determinants of health rather than as fixed group properties: some cognitive measures vary across menstrual-cycle phases within the same individuals [30], gender-linked occupational and economic experiences are associated with late-life cognitive outcomes [31], and in settings where educational and social opportunity differ sharply by gender, lower cognitive scores in women accompany those disparities in educational attainment and socioeconomic position [32]. We therefore frame the present four-group comparison descriptively, as a characterisation of performance in a specific clinical and cultural setting, and not as a test of which category cognition resembles.

### 4.3. Visuospatial Functioning and Executive Control in Context

Visuospatial abilities—assessed via the Judgment of Line Orientation Test and multiple indices of the Rey–Osterrieth Complex Figure Test showed the most robust group effects. These domains are among the most frequently examined in sex/gender research, with meta-analytic evidence supporting small average male advantages in certain spatial tasks alongside considerable heterogeneity [28,29].

Findings in transgender samples, however, have been notably inconsistent.

Neuroimaging and cognitive studies focusing on mental rotation and related paradigms suggest that observed patterns cannot be reduced to a binary male–female distinction and may vary according to task demands, developmental timing, and psychosocial context [24,33]. Although mental rotation was not assessed in the present study, the observed differences in visuospatial perception, organization, and recall similarly point toward domain-specific and context-sensitive influences.

Notably, performance on the Rey–Osterrieth Complex Figure Test depends not only on visuospatial processing but also on planning, organizational strategy, and cognitive–affective load. Under conditions of chronic stress, these higher-order control processes may be particularly vulnerable. Thus, lower performance on complex visuospatial tasks in transgender participants should not be interpreted as evidence of reduced capacity, but rather as a potential manifestation of stress-related cognitive load and resource depletion in evaluative settings.

### 4.4. Executive Functions, Stress Sensitivity, and Minority Stress

Group differences were observed for executive function–dependent measures of the Trail Making Test (TMT-B and B–A difference), but not for basic processing speed (TMT-A). In cisgender populations, sex/gender differences in executive control are generally small and inconsistent, as demonstrated in meta-analytic studies [34,35]. Executive functions, however, are well known to be sensitive to stress, anxiety, sleep disruption, and sustained cognitive vigilance.

Within this framework, the observed pattern—cisgender women showing the fastest executive performance, followed by cisgender men, with both transgender groups showing longer completion times—suggests that psychosocial factors may exert a stronger influence than identity classification per se. Minority Stress Theory provides a compelling explanatory lens, proposing that chronic exposure to stigma and discrimination consumes cognitive resources and impairs executive control over time [15,16].

In addition, the concept of stereotype threat may be relevant. Transgender individuals undergoing cognitive assessment in a sociopolitical environment characterized by negative public discourse may experience heightened concern about confirming stigmatizing assumptions, which in turn can transiently impair performance on demanding cognitive tasks. This mechanism offers a plausible, non-pathologizing account of the executive function differences observed in this study.

Notably, structural and minority stress–related factors may exert effects on cognitive functioning that are independent of, and in some contexts comparable to, traditional sociodemographic predictors such as educational attainment [15].

Within this framework, minority stress may represent a cumulative cognitive burden that interacts with—but is not reducible to—educational background. Although our study did not directly quantify minority stress, population-based evidence illustrates that identity-linked stressors can show meaningful associations with cognitive outcomes even after accounting for education. For example, in a large cohort of sexual minority older adults, minority stress (sexual-orientation–related negative social experiences) was associated with poorer fluid intelligence after adjustment for age, sex, and education (B = −2.116, *p* = 0.016; partial η^2^ = 0.044), an effect size comparable to—and in that model larger than—several education-level contrasts [36].

Longitudinal work similarly links everyday discrimination to worse subsequent performance across cognitive domains independent of sociodemographic factors, with particularly durable direct effects on executive functioning and visuoconstruction [37]. These findings support interpreting our executive and complex visuospatial differences through a stress-related cognitive load framework rather than a deficit model tied to identity categories.

### 4.5. Working Memory and the Role of Assessment Context

No significant differences in digit span performance were observed across the four gender groups, despite aggregated transgender–cisgender analyses indicating higher backward and total digit span scores among transgender participants. Statistically, this discrepancy is plausible: subgroup analyses with modest sample sizes—particularly among transgender women (*n* = 14) and cisgender women (*n* = 13)—are vulnerable to Type II error, potentially obscuring small-to-moderate effects.

From a clinical perspective, the digit span findings are noteworthy. Digit span tasks are less culturally and linguistically loaded than tasks requiring complex visuoconstruction or semantic retrieval. Moreover, in the present assessment sequence, digit span was administered after rapport had been established with the examiner. The combination of a supportive testing environment and reduced situational anxiety may have allowed working memory performance to emerge more clearly, illustrating how rapport building and order effects can partially counteract stress-related cognitive burden. This observation aligns with principles of affirmative neuropsychological practice [13,14].

### 4.6. Verbal Fluency, Language, and Cultural Context

The absence of significant four-group differences in semantic and phonemic verbal fluency warrants careful interpretation. Verbal fluency is among the domains most sensitive to language structure, educational experience, and cultural norms, and findings from Western, English-speaking samples cannot be assumed to generalize directly to other linguistic contexts.

Turkish is a morphologically rich language, with agglutinative structure and distinct lexical organization. Semantic category familiarity, word frequency distributions, and retrieval strategies differ substantially from those assumed in Western norms. Recent studies in Turkish-speaking populations have documented wide variability in semantic verbal fluency performance and scoring practices, underscoring the need for culturally calibrated interpretation [38].

Accordingly, the lack of robust group differences in verbal fluency in the present study may reflect substantial overlap across groups and interactions between linguistic structure, education, and sociopolitical contexts. This finding further cautions against uncritical application of Western normative frameworks in non-Western settings.

### 4.7. Gender-Affirming Hormone Therapy and Neurocognition

Exploratory analyses did not reveal significant neuropsychological differences between transgender participants receiving gender-affirming hormone therapy and those who were not. While the modest size of the transgender women subgroup limits statistical power, the absence of detectable hormone-related effects is consistent with systematic reviews and meta-analyses reporting no overall adverse cognitive impact of hormone therapy in transgender adults [12,25,39]. However, these analyses may have been underpowered, particularly given the small size of subgroups not receiving hormone therapy, and therefore should not be interpreted as evidence of absence of an effect. Additionally, given the number of exploratory comparisons conducted, these findings should be interpreted with caution and considered preliminary.

From a neurobiological standpoint, the relationship between circulating hormone levels and cognition is complex. Cognitive effects may depend not only on serum concentrations but also on receptor sensitivity, regional receptor distribution, timing and duration of exposure, and interactions with psychosocial stressors. Under conditions of chronic minority stress, subtle hormone-related cognitive effects may be masked or overridden by stress-related neuroendocrine mechanisms. Moreover, cross-sectional designs are inherently limited in detecting such nuanced changes, which are more appropriately examined in longitudinal, within-person studies [9].

In our dataset, the absence of associations for GAHT status, GAHT duration, and estradiol—and the presence of only a single weak correlation between testosterone and ROCF delayed recall completion time—suggest that any hormone–cognition relationships, if present, are likely to be subtle, non-linear, or contingent on moderators such as timing of exposure, receptor sensitivity, and chronic stress physiology. Given the exploratory analyses and multiple comparisons, this isolated association should be interpreted conservatively and prioritized for replication in longitudinal, within-person designs.

### 4.8. Sociopolitical Context and Non-Pathologizing Interpretation

The sociopolitical context of Türkiye constitutes a critical backdrop for interpreting these findings. Transgender individuals face not only interpersonal discrimination but also a broader political climate marked by hostile official rhetoric, legal uncertainty, and constraints on civil society and academic expression [18,20,21]. In such an environment, cognitive assessment may occur under conditions of heightened vigilance and perceived threat.

Rather than framing observed differences as indicators of cognitive deficit, our findings are more appropriately understood as reflecting stress-related cognitive load within a structurally stigmatizing context. Beyond interpretation, these findings also raise broader questions about how neuropsychological assessment norms are constructed.

Most cognitive test norms are derived from cisgender populations and may not fully capture the sociocultural and structural contexts in which transgender individuals are assessed. In the longer term, accumulating datasets that include transgender participants from diverse cultural settings may contribute to the development of more inclusive normative frameworks and more equitable cognitive assessment practices.

A further observation deserves emphasis. Although approach, refusal, and completion rates were not systematically recorded and cannot therefore be reported, the completion of a 60–90 min neuropsychological battery by 53 transgender adults demonstrates the feasibility of clinic-embedded assessment in this setting, and the transgender sample is, to our knowledge, the largest assessed with a full battery in Türkiye. Embedding assessment within an established multidisciplinary care pathway may facilitate participation relative to stand-alone research encounters, although this study cannot test that possibility. Prospective studies should formally document recruitment, refusal, and completion metrics, which would allow the feasibility of such designs to be evaluated directly.

### 4.9. Strengths and Limitations

Strengths of this study include its four-group analytic design, standardized neuropsychological assessment, age- and education-matched cisgender controls, and conduct within a multidisciplinary gender-affirming care clinic. To our knowledge, this represents the first standardized neuropsychological dataset from an adult transgender sample in Türkiye.

Recruiting and retaining a clinically evaluated transgender sample of this size for standardized neuropsychological assessment can be particularly challenging in many non-Western settings. The present cohort therefore represents a meaningful contribution obtained under real-world clinical conditions.

Limitations include the cross-sectional design, imbalanced subgroup sizes—particularly the smaller number of transgender women—and the absence of direct measures of minority stress, sleep, and affective symptoms. These factors may have limited sensitivity to subtle effects and increased the risk of Type II error in subgroup analyses. Menstrual cycle phase at the time of testing was not recorded in cisgender women, and its potential influence on visuospatial and executive performance could therefore not be examined. In addition, the relatively small sample sizes in some subgroups—particularly transgender women and cisgender women—may have limited statistical power, increasing the risk of Type II error and reducing sensitivity to detect small-to-moderate effects. The lower representation of transgender women should be interpreted in light of the sociopolitical context of Türkiye, where transgender women are exposed to more severe and visible forms of stigma, violence, and social exclusion. This environment may constrain access to specialized clinical services and reduce willingness or ability to participate in lengthy neuropsychological assessments, introducing a context-specific sampling bias related to safety and feasibility rather than study design. Additionally, the clinic-based sample reflects individuals most likely to seek gender-affirming medical interventions, which may limit generalizability to non-binary or non-clinical populations.

Despite these modest subgroup sizes, this dataset contributes a rare, non-Western clinical cohort to a literature that often depends on multi-center aggregation. As such, these data are highly valuable for future pooled analyses and cross-cultural syntheses aimed at improving the generalizability of neurocognitive evidence in transgender health research. Furthermore, because neuropsychological tests are embedded in linguistic and cultural norms, transgender and gender-diverse populations must be included in future normative and validation work. Establishing context-appropriate reference data—particularly in non-Western settings—is an important longer-term goal to support more equitable cognitive assessment. This will help clinicians interpret performance without resorting to deficit-oriented framings and ultimately improve the clinical utility of cognitive evaluation in gender-affirming care. Future studies should incorporate direct measures of minority stress, perceived discrimination, and psychosocial burden in order to more precisely evaluate their relationship with neurocognitive performance.

## 5. Conclusions

In this Turkish clinical sample, the observed distributions were domain-specific and substantially overlapping, and should not be interpreted as indicating correspondence between cognitive performance and either gender identity or SAAB. Instead, domain-specific differences were most evident in visuospatial and executive functions, while working memory and verbal fluency showed substantial overlap across groups. These findings support a context-sensitive, non-pathologizing interpretation that integrates sociocultural, methodological, and stress-related determinants, and underscore the need for longitudinal, cross-cultural research incorporating direct measures of minority stress and neurobiological change.

## Figures and Tables

**Table 1:** Socio-demographic characteristics by gender group.

Variable	Trans Women (*n* = 14)	Trans Men (*n* = 39)	Cis Women (*n* = 13)	Cis Men (*n* = 15)	*p*
Age, years	22.1 (3.6)	27.2 (6.1)	27.9 (8.3)	26.2 (5.7)	0.022
Education, years	12.9 (1.2)	14.0 (3.5)	14.5 (3.4)	13.5 (3.2)	>0.05
Right-handed, *n* (%)	12 (85.7)	34 (87.2)	11 (84.6)	12 (80.0)	>0.05

Note. Values are presented as mean (SD) unless otherwise indicated. Continuous variables were analyzed using the Kruskal–Wallis test, and categorical variables using the chi-square test. TW = trans women; TM = trans men; CW = cis women; CM = cis men.

**Table 2:** Neuropsychological test performance by gender group, presented by cognitive domain.

Test	Trans Women (*n* = 14)	Trans Men (*n* = 39)	Cis Women (*n* = 13)	Cis Men (*n* = 15)	*p*
Visuospatial abilities					
JLO total score	23.5 (4.7) a Mdn = 24	22.9 (4.5) a Mdn = 23	25.9 (4.4) ab Mdn = 27	27.5 (2.9) b Mdn = 28	<0.001
ROCF copy	28.4 (5.5) a Mdn = 28	30.1 (4.9) a Mdn = 30	33.5 (7.2) b Mdn = 36	34.9 (2.3) b Mdn = 36	<0.001
ROCF immediate recall	12.9 (5.8) a Mdn = 12.5	14.4 (5.5) a Mdn = 13	19.9 (8.1) b Mdn = 22	21.1 (6.5) b Mdn = 20	<0.001
ROCF delayed recall	12.9 (7.2) a Mdn = 12	14.1 (6.4) a Mdn = 14	19.6 (7.5) b Mdn = 20	19.5 (7.4) b Mdn = 18	0.008
Executive function and processing speed					
TMT-A (s)	39.2 (20.6)	31.4 (12.8)	36.4 (21.2)	29.1 (10.6)	>0.05
TMT-B (s)	102.9 (34.1) a	86.6 (30.5) ab	69.2 (25.5) b	78.1 (31.8) ab	0.048
TMT B–A (s)	63.6 (29.0) a	54.0 (27.4) a	32.8 (14.3) b	49.0 (24.9) ab	0.014
Working memory					
Digit span forward	5.4 (1.3)	5.9 (1.6)	5.5 (1.2)	5.9 (1.7)	0.363
Digit span backward	4.3 (1.1)	4.9 (1.6)	4.3 (1.1)	4.9 (1.6)	0.161
Total digit span	9.7 (2.3)	10.8 (3.1)	9.7 (2.3)	10.8 (3.1)	0.202
Verbal fluency					
Semantic fluency (animals)	20.9 (6.2)	19.5 (4.4)	21.0 (6.3)	24.1 (5.3)	0.071
Phonemic fluency (K–A–S total)	29.9 (10.6)	30.7 (12.8)	27.9 (10.4)	27.1 (11.9)	0.737

Note. Values are presented as mean (SD); medians (Mdn) are given for the visuospatial measures. Group comparisons were performed using the Kruskal–Wallis test and are descriptive rather than confirmatory. JLO = Judgment of Line Orientation; ROCF = Rey–Osterrieth Complex Figure; TMT = Trail Making Test; B–A = difference score. Values with different superscript letters (a, b) within a row are significantly different at *p* < 0.05 based on Dunn’s post-hoc tests with Bonferroni correction.

**Table 3:** Gender-affirming hormone therapy (GAHT) status, treatment duration, and serum hormone concentrations among transgender participants.

Group	GAHT Status	*n*	Duration (Months)	Total Testosterone (µg/L)	Estradiol (pg/mL)
Trans women	Receiving	11	17.0 (9.2)	0.98 (1.84)	86.8 (81.4)
Trans women	Not receiving	3	—	2.88 (1.20)	74.5 (75.4)
Trans men	Receiving	21	27.7 (19.4)	6.48 (4.24)	72.2 (48.2)
Trans men	Not receiving	18	—	0.58 (0.46)	97.8 (59.9)

Note. Values are presented as mean (SD). Duration is reported only for participants receiving GAHT. Hormone units are reported as provided in the clinical laboratory records. TW = trans women; TM = trans men.

## Data Availability

The data that support the findings of this study are available from the corresponding author upon reasonable request. The data are not publicly available due to ethical restrictions and the sensitive nature of the data.
